# Cancer Metastasis: A Therapeutic Target

**DOI:** 10.1155/2019/7907282

**Published:** 2019-08-07

**Authors:** Peramaiyan Rajendran, Wei-Ting Chao, Esaki M. Shankar, Ekambaram Ganapathy, Kalayarasan Srinivasan

**Affiliations:** ^1^Department of Biological Sciences, College of Science, King Faisal University, Hofouf, Al-Ahsa 31982, Saudi Arabia; ^2^Department of Life Science, Tunghai University, Taichung, 40704, Taiwan; ^3^Division of Infection Biology and Medical Microbiology, Department of Life Sciences, Central University of Tamil Nadu, Neelakudi, Thiruvarur 610 005, Tamil Nadu, India; ^4^Department of Radiation Oncology, Division of Molecular Oncology, University of California at Los Angeles (UCLA), Los Angeles, CA 90095, USA; ^5^Division of Molecular Radiation Biology, Department of Radiation Oncology, University of Texas Southwestern Medical Center, Dallas, TX, USA

Metastasis to vital organs from cancers of the liver, breast, lung, colon, melanoma, and prostate accounts for ~90% of all cancer-related deaths. Investigators have published a wealth of data on basic science and clinical medicine documenting metastases and their prognostic significance, pathobiological mechanisms, therapeutic targets, and diagnostic advances. Most of the work has been dedicated to understand the importance and significance of cancer metastasis and the clinical therapeutic proteins targeted for potential translational implications. Consequently, given that, evidences pertinent to such measures have set the stage for clinicians and basic medical scientists to advance from the bench to the bedside, and the Journal of Oncology has set out to publish a special issue devoted to a topic titled “Cancer Metastasis: A Therapeutic Target.” The result is a collection of ten outstanding articles submitted by investigators representing ten countries across North America, Europe, and Asia. In all cases, the methods section of each manuscript included a statement documenting that the clinical investigations were performed following institutional review board approval and/or that informed consents from patients or their legal representatives were secured before setting out for the respective investigations.

S. Sundararajan et al. (2019), based in USA, in the paper titled “Sequential Interventional Management of Osseous Neoplasms via Embolization, Cryoablation, and Osteoplasty,” have demonstrated that combination therapy has the potential to transform into an effective mainstay of treatment paradigm in the palliative care of osseous neoplasms to improve the quality of life of individuals. A. Trebinska-Stryjewska et al. (2019), from Poland, in the paper titled “Cytoplasmic HAX1 Is an Independent Risk Factor for Breast Cancer Metastasis,” have established that HAX1 localization is important for the prediction of metastatic relapse and that cytoplasmic but not nuclear HAX1 is an independent risk factor for breast cancer metastasis. S. M. Messerli et al. from USA in their article titled “Use of Antimetastatic SOD3-Mimetic Albumin as a Primer in Triple Negative Breast Cancer” support the hypothesis that PNA works through the inhibition of extracellular superoxide/ROS production leading to the conversion of 4T1 cells from a metastatic tumorigenic state to a cytostatic state. Their findings advocate potential future clinical trials using PNA as an antimetastatic SOD3-mimetic drug to increase overall survival in TNBC patients.

A. Bisgin and Colleagues (2019) from Turkey in their article titled “Interaction of CD200 Overexpression on Tumor Cells with CD200R1 Overexpression on Stromal Cells: An Escape from the Host Immune Response in Rectal Cancer Patients” have clearly shown that tumor-stroma communication through CD200 and its receptor interaction is selected in patients with high risk of relapse. High levels of these molecules support instigation of the far and local metastatic nest that provides solid ground for metastasis. S. H. Kim et al. (2019) from South Korea in the paper titled “The De Ritis and Neutrophil-to-Lymphocyte Ratios May Aid in the Risk Assessment of Patients with Metastatic Renal Cell Carcinoma” have demonstrated that the overall survival and predictive ability were increased when NLR and DRR markers were added to established Heng or mMSKCC risk models in patients with mRCC treated with first-line targeted therapy. E. Dirican and E. Kiliç (2019) from Turkey in the paper titled “A Machine Learning Approach for the Association of ki-67 Scoring with Prognostic Factors” investigated the way of clustering of prognostic factors by ki-67 score using a machine learning approach and multiple correspondence analysis. They have found that low scores of ki-67 correlate with early-stage disease and high scores with advanced disease suggesting that 14% threshold value is crucial for ki-67 score. S. Destek and V. O. Gul in their article titled “S100A4 May Be a Good Prognostic Marker and a Therapeutic Target for Colon Cancer” have established the role of S100A4 in the prognosis of colon cancer and its prognostic significance. Their work provides a unique perspective on the published clinical significance of S100A4 in colon cancer prognosis.

A series of three review papers, one from Portugal titled “Liver Metastases and Histological Growth Patterns: Biological Behavior and Potential Clinical Implications—Another Path to Individualized Medicine?” by R. I. Oliveira et al. (2019), one from China titled “Resection of Liver Metastases: A Treatment Provides a Long-Term Survival Benefit for Patients with Advanced Pancreatic Neuroendocrine Tumors: A Systematic Review and Meta-Analysis” by X. Yu et al. (2019), and one from China as well titled “iRGD, A Promising Peptide for Cancer Imaging and Potential Therapeutic Agent for Various Cancers” by H. Zuo (2019), have clearly examined several diverse therapeutic targets on cancer metastasis. Each work provides a unique perspective on published clinical trials on the importance and therapeutic targets associated with cancer metastases. Uniformly, all authors highlight both the promise and the challenges faced by clinicians and basic medical scientists in the emerging field of cancer metastasis. Their manuscripts identify the critical need for additional prospective, randomized controlled clinical trials evaluating metastasis. In summary, this special issue provides a snapshot of the status of cancer metastasis and clinical trials carried out in the area from across the globe. We hope that the articles published herein will provide a benchmark for future meta-analyses evaluating a far greater body of clinical evidence regarding the safety and efficacy of cancer metastasis therapies, and we sincerely trust that the articles published in this special issue will amuse the audience of the Journal of Oncology that has dedicated itself to ending the menace that indeed is awaiting its end.

